# Process Optimization for Metal-Contact Etching in 3D Integration Devices

**DOI:** 10.3390/mi16121354

**Published:** 2025-11-28

**Authors:** Sung Gyu Pyo

**Affiliations:** School of Integrative Engineering, Chung-Ang University, 84, Heukseok-ro, Dongjak-gu, Seoul 06974, Republic of Korea; sgpyo@cau.ac.kr

**Keywords:** 3D integration, super-contact, contact etch, metal-1 contact

## Abstract

This study investigates a metal-contact etching process that differs from conventional device contact etching by focusing on the film-stack configuration and the associated super-contact etching characteristics. Because metal-contact etching is closely linked to both physical profiles and electrical performance, evaluating a single parameter provides limited insight; thus, the physical profile characteristics of metal-contact etching and 3D-integrated super-contacts were comprehensively examined. In the first-step etch, the target depth in the wafer left region was approximately 2365 Å, and the bottom surface exhibited a desirable rounded profile. Following the removal of liner TEOS and nitride, the stopping margin was evaluated under three conditions: (1) metal-contact etching with a ~22 s target reduction, (2) a CMOS image-sensor baseline incorporating an interlayer-dielectric-reduction scheme, and (3) a high-selectivity condition achieved by increasing the C_5_F_8_/O_2_ ratio with a reduced etch target. Under all three conditions, the bit-line contact (BLC) nitride experienced punch-through. To address this limitation, a three-step etch sequence was implemented, in which the first two steps achieved the required etch depth and the final step utilized a high-selectivity over-etch to secure a sufficient stopping margin. This approach demonstrated robust process windows, favorable CD control, and reliable nitride stopping performance, thereby establishing a practical methodology for stable super-contact etching in advanced 3D-integrated logic applications.

## 1. Introduction

As the semiconductor industry continues to pursue high-performance computing solutions that maximize miniaturization and efficiency, system-on-chip (SoC) and system-in-package (SiP) technologies have emerged as key technological paradigms [1]. SoCs integrate the entire components of a computer or other electronic system into a single integrated circuit (IC), optimizing performance and reducing power consumption [2,3]. SiP technology complements SoCs by packaging multiple ICs into a single module, thus offering flexibility in combining various technologies and functions. These approaches play a critical role in meeting the ever-increasing demands for the miniaturization and integration of electronic devices [4,5,6].

The continuous scaling toward ultra-dense device structures has accelerated the development of 3D integration, which provides a fundamental solution to the physical and performance limitations of planar architectures. By vertically stacking and interconnecting multiple layers, 3D integration increases device density, enhances performance, and reduces power consumption [7,8,9,10].

The transition to 3D integrated devices increases the demand for reliable process technologies. Key processes include metal-1 (M1) contact etching, super-contact etching, Super-Via filling, wafer bonding, and wafer thinning. These processes are crucial for ensuring the structural integrity and electrical performance of the devices [11,12,13]. Challenges such as increased signal delay, crosstalk, and electromagnetic interference further emphasize the importance of precise etch control and robust interconnect formation [14,15].

Despite significant progress, existing device-contact etching schemes, including those used for conventional 180 nm logic (L180), exhibit intrinsic limitations when applied to 3D-integration film stacks. These limitations include insufficient stopping margin, degraded bottom selectivity, increased CD variability across wafer positions, and incompatible ILD film-stack architectures (ONO vs. ONONO). As a result, one-step M1 contact etching cannot achieve stable borderless-contact formation in 3D integration. This constitutes a major technical gap in current manufacturing flows.

To address these limitations, this study introduces and evaluates a three-step M1 contact etch scheme specifically designed for 3D-integration ILD structures. The proposed approach is motivated by the need to (i) restore adequate stopping margin within thick ONONO stacks, (ii) suppress punch-through of the BLC nitride layer, (iii) minimize CD bias across wafer positional regions (LBCTR), and (iv) optimize selectivity through tailored C_5_F_8_, C_3_F_6_, and C_4_F_8_ fluorocarbon chemistries. Unlike conventional one-step etch flows, the three-step method provides sequential control over the main etch, profile tuning, and high-selectivity over-etch regions, enabling improved robustness against wafer-position-dependent loading effects.

## 2. Experimental Procedure

**Figure 1:**
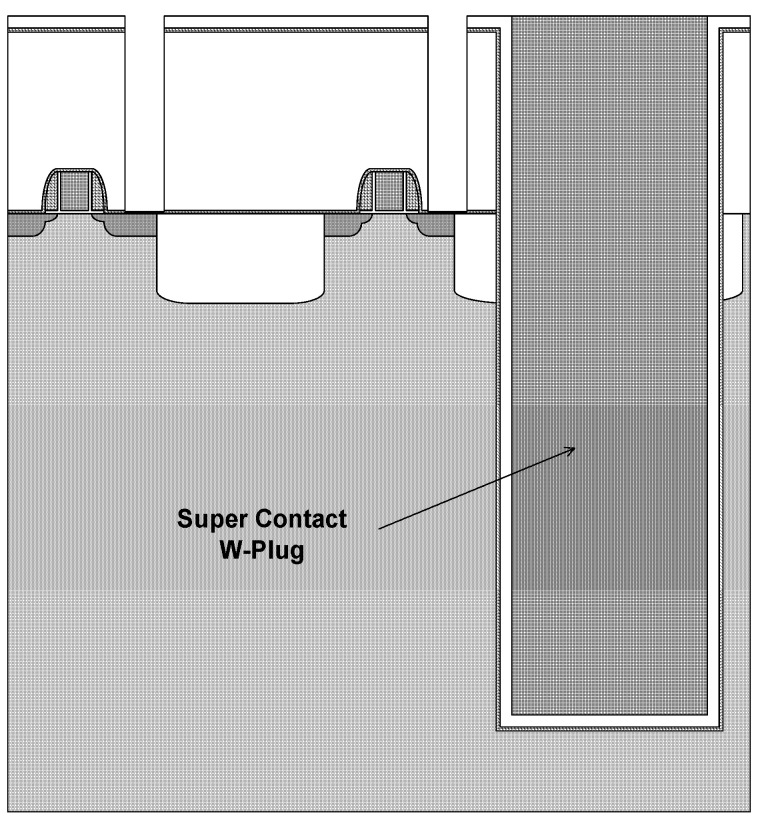
ILD structure in the 3D integration scheme.

Borderless contact is used as the baseline scheme for 180 nm process integration. In 3D integration, however, the ILD configuration differs significantly because liner nitride (500 Å) and TEOS (1500 Å) are deposited after the Super-Via etch, forming an ONONO stack (Figure 1). This modified ILD structure prevents the direct application of the one-step M1CT etch used in the L180 flow and therefore requires a dedicated multistep M1CT etch strategy.

The complete 3D-integration M1CT ILD process flow is summarized in Table 1. The sequence begins with the deposition of 300 Å BLC nitride, followed by 3500 Å BPSG and 12,000 Å TEOS. After ILD CMP, the remaining ILD thickness is approximately 8000 Å. Subsequently, Super-Via ILD and Si etching are performed, after which a 500 Å liner nitride and 1500 Å liner TEOS are deposited. Tungsten is then deposited to 1 μm and planarized by CMP, after which the M1CT photo/etch step is carried out.

**Figure 2:**
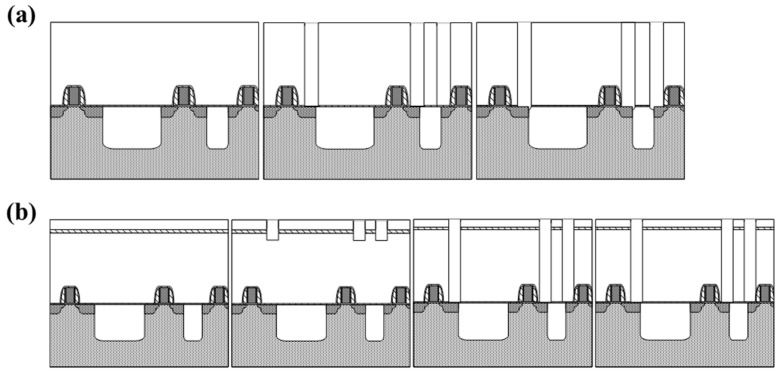
(**a**) 180 nm M1 contact ILD structure etch scheme and (**b**) 3D integration M1CT etch scheme.

As illustrated in Figure 2, the Super-Via functions as the vertical interconnect between bonded wafers. During backside thinning and Super-Via exposure, the liner nitride and TEOS films serve as protective stoppers, preventing mechanical or chemical damage to the W-plug.

Cross-sectional ILD thicknesses were measured using scanning electron microscopy (SEM, SIGMA 360, Carl Zeiss, Oberkochen, Germany) after cleaving the wafers and preparing cross-section samples. In addition, fluorocarbon (F/C) polymer residues generated during etching were analyzed using X-ray photoelectron spectroscopy (XPS, K-Alpha^+^, Thermo Fisher Scientific, Waltham, MA, USA).

## 3. Results

### 3.1. 3D Integration M1CT ILD Structure Analysis

The ILD1 (Interlayer dielectric 1) (Boro phospho silicate glass (BPSG) + TEOS) thickness was confirmed to be 7500 Å, which is about 500 Å lower than the target. This is because hundreds of angstroms of oxide were lost during Buffered Oxide Etch (BOE) cleaning after the Super-Via Si etch. For more accurate target setting, the loss during BOE cleaning should be considered during ILD CMP. The ILD2 (interlayer dielectric 2) (Liner nitride + Liner TEOS) deposited after the Super-Via Si etch had a loss of about 100 Å after tungsten CMP, which was approximately 1900 Å. Therefore, the total ILD thickness excluding the BLC nitride application was approximately 9400 Å, which is about 1000 Å higher than that in L180.

### 3.2. Step-Etching Condition for Liner TEOS & Nitride Etch

Regarding the first-step etch recipe, applying the L180 baseline conditions (C_5_F_8_/O_2_/Ar) results in etching terminating at the liner nitride layer. This is because the L180 chemistry is optimized for high SiO_2_/Si_3_N_4_ selectivity: fluorocarbon species readily etch oxide while the nitrogen-rich Si_3_N_4_ surface promotes polymer stabilization, significantly reducing its etch rate [16,17,18]. As a result, the liner nitride functions as an effective intrinsic stop layer. Hence, for 3D-integration M1CT etching, the first step requires a non-selective condition that eliminates nitride stopping behavior and ensures complete liner removal. In our experiment, we used CHF_3_/O_2_/Ar chemistry (nitride-to-oxide selectivity: 1.22), which was used in storage node contact (SNC) etching, for the first-step etch. Compared with those in other contact-etch processes, the liner TEOS and nitride layers were thinner; moreover, oxide CMP was not performed. Hence, the over-etch target was set at approximately 20–30% to control the remnant ILD after the first-step etch; we targeted roughly 8500 Å or 6500 Å under the ILD reduction scheme. Based on an etch rate evaluation, the TEOS in the ILD2 layer was assumed to become thinner by about 100 Å after Super-Via tungsten CMP, which left approximately 1400 Å; the liner nitride was deposited at 500 Å with minimal error. Table 2 summarizes the etch targets. Specifically, the liner TEOS and nitride etch for ILD2 required about 13.5 and 3.9 s, respectively, while the TEOS for ILD1 was over-etched for 5.6 s, which resulted in a thickness of about 600 Å.

Plasma characteristics vary with pressure and gap size. For pressures above 40 mT, the ion energy remains largely constant, and the ion flux is dominant. Below 15 mT, the ion energy and flux are both important. An increase in the gap reduces the plasma density and vice versa [19]. However, as the plasma density changes by less than an order of magnitude with gap adjustment, the gap size should be adjusted primarily to control uniformity.

**Figure 3:**
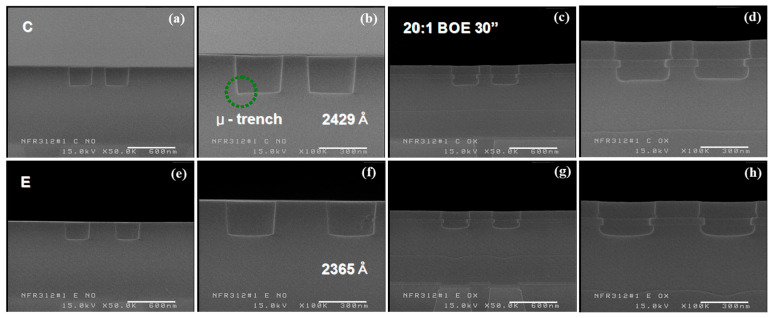
Results of 3D integrated M1CT phase 1st etch target and profile evaluation: (**a**,**e**) etch profiles at the center and edge; (**b**,**f**) corresponding magnified views; (**c**,**g**) post-etch morphologies after 30 s in 20:1 BOE; (**d**,**h**) magnified views of (**c**,**g**).

Under the conditions listed in Table 2, a 23-s target etch was performed, and the etch profiles were analyzed by wafer region (Figure 3). On the left side, the etch target was about 2365 Å, roughly 140 Å below the expected value; however, the bottom surface showed a favorable rounded profile. In contrast, the wafer center had an etch target of about 2430 Å, closely matching the calculated value, with minimal micro-trench formation at the sidewall.

Table 3 summarizes the residual ILD thickness after the first etch, based on the ILD2 etch target. The center region showed an etch target about 60 Å higher; however, this value was within the measurement error and was considered similar. The ILD2 thickness at the center was about 100 Å greater than that at the edge, which indicates that the actual over-etch target was roughly 3% higher at the edge. Despite this inverse relationship between the ILD2 thickness difference and the etch uniformity, no significant difference in the remnant ILD thickness was observed between the center and the edge after the first-step etch.

The stopping margin for bit-line contact (BLC) nitride was evaluated using L180 M1CT etch recipes following the removal of the initial liner TEOS and nitride. Evaluations were conducted under three conditions: (1) a target reduction of approximately 22 s under the L180 M1CT etch, (2) the standard M1CT process with the ILD reduction scheme, and (3) an increased C_5_F_8_/O_2_ ratio for improved selectivity and a slightly reduced etch target. Despite exhibiting favorable etch stop margins under the L180 scheme, which refer to the allowable etch window required to avoid over-etching into the underlying metal contact and thus prevent junction damage, all conditions nonetheless resulted in BLC nitride punch-through. As summarized in Table 4, the over-etch percentages were 24.5% at the wafer center and 27.8% at the edge. This outcome is likely due to differences in the cathode temperature and polymer deposition state at the etch front surface. Specifically, the M1CT tool operates at a bottom electrode temperature of −10 °C, which is unsuitable for BLC etching requiring strong bottom selectivity. The sticking probability coefficient of fluorocarbon polymers is typically 0.2–0.5 and is inversely proportional to temperature [20,21,22], causing excessive sidewall polymer accumulation and reducing reactive species transport to the etch front. This imbalance promotes bottom etching relative to the sidewalls under ion bombardment, thereby degrading nitride stopping performance and leading to punch-through. In contrast, deep-contact processes for dynamic random access memory (DRAM) necessitate low bottom-electrode temperatures to encourage sidewall polymer deposition, prevent bowed profiles, and suppress etchant radical reactions at the sidewall instead of the hole bottom, where etching is desired. Notably, the smooth etching at the bottom rather than the sidewall at low temperatures is due to the higher ion-assisted etch yield at the bottom. For these reasons, low bottom-electrode temperatures are unsuitable for borderless contact in logic devices, and temperatures of 20 °C or higher are preferable in the long term. Consequently, because a 300 Å BLC nitride etch stop margin was insufficient under these conditions, a three-step etch recipe was evaluated: etching to appropriate targets in the first and second steps, followed by high-selectivity over-etching in the third step.

### 3.3. Second-Step Etch Rate Process Condition

Under these conditions, several factors should be considered when setting the second etch parameters. First, since the third etch conditions may cause etch stopping at about 4000 Å or more, maximizing the etch target in the second step is important. Second, if the ILD thickness is reduced by ILD deposition or CMP, etching could occur down to the BLC nitride in the second step; therefore, variations in ILD deposition and CMP must be considered. Accordingly, in the second-step etch, we targeted the removal of most of the ILD by applying conditions with relatively low selectivity to maintain the final inspection critical dimension (FICD). The evaluation conditions are presented in Table 5.

**Figure 4:**
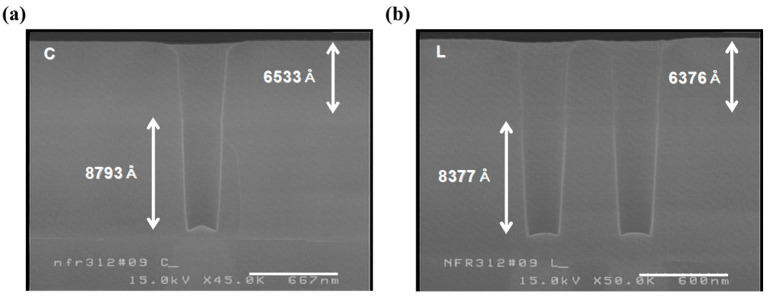
Second-step etch-rate evaluation using C_4_F_8_/O_2_/Ar. SEM images from the (**a**) wafer center regions and (**b**) left regions show etch targets of ~8800 Å and ~8400 Å, confirming consistent PR loss and stable etch behavior across the wafer.

To verify both the photo resist (PR) profile and loss, specimens for cross-sectional analysis were prepared by etching and stripping the PR film. Since the experiment focused on confirming the etch target, only a gold coating was sputtered for 80 s without BOE treatment before scanning electron microscopy (SEM) analysis. BPSG and plasma-enhanced tetraethyl orthosilicate (PE-TEOS) have different etch rates due to differences in their physical properties. To ensure greater accuracy, determining the etch rates for each film type is the standard practice. However, due to the limited availability of test wafers, the approximate etch target and rate were determined using a bilayer stack (Figure 4). The wafer center had a target of about 8800 Å, including the first step. Excluding the first step, the etch target and rate were 6364 Å/min and 6942 Å/min, respectively. On the wafer’s left side, the etch target was about 400 Å lower than that at the center, with the corresponding values being 6012 Å and 6559 Å/min, respectively. The PR loss did not vary significantly among regions.

### 3.4. Stopping Conditions Based on Third-Step Etch Conditions

After the second-step etch, we aimed to identify the conditions for each gas chemistry that would allow stopping on BLC nitride. Using the two main etchants, C_4_F_8_ and C_5_F_8_, which provide high selectivity for nitride with increased polymer stiction [18], we selected the third-step etch conditions that would enable etch stopping while achieving a target thickness of several thousand angstroms. This approach ensured that a stopping margin under oxide etch conditions could be achieved with the applied bottom electrode settings (Table 6).

When etching with a 2600 Å target under 18C_4_F_8_/8O_2_/420Ar conditions, which provide a selectivity of about 20:1 at +20 °C, only around 150 Å of BLC nitride should be lost before stopping, unless the setting is a baseline condition. However, a cross-sectional analysis of the SRAM area of wafer1, cut along the *X*-axis, revealed BLC nitride punch-through in all regions. Because the size of the mass flow controller (MFC) fixed to the O_2_ gas line was 50 sccm, maintaining it below 5 sccm was not desirable. Thus, securing a stopping margin under C_4_F_8_/O_2_/Ar conditions was deemed infeasible. In contrast, wafer2 under 16C_5_F_8_/9O_2_/800Ar conditions stopped at 300 Å of BLC nitride.

**Figure 5:**
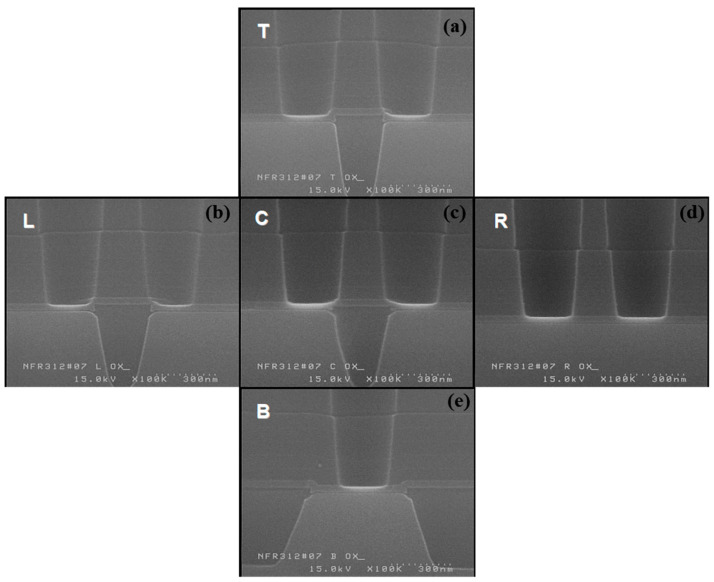
Stopping-margin assessment under the third-step 16C_5_F_8_/9O_2_/800Ar condition. All wafer regions show controlled BLC nitride loss (~150 Å max) and successful etch stopping on the 300 Å nitride layer: (**a**) top, (**b**) left, (**c**) center, (**d**) right, and (**e**) bottom.

Because the third-step conditions were prone to etch stopping, the stopping margin and BLC nitride loss were examined across the wafer via a cross-sectional analysis (Figure 5). While approximately 150 Å of BLC nitride loss was measured in the highest-etch-rate central region, the process demonstrated sufficient stopping margin retention.

**Figure 6:**
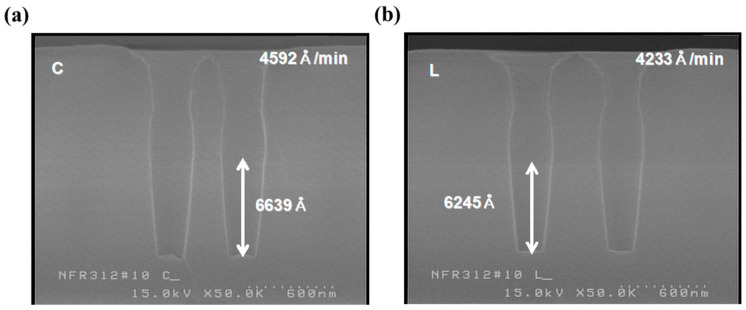
Results for etch target after first-step + third-step (16C_5_F_8_/9O_2_/800Ar) etch: (**a**) center; (**b**) left.

To verify the third-step etch target for C_5_F_8_/O_2_/Ar, the third-step etch was performed after applying the first-step recipe, and the etch rate and target were checked for each wafer region (Figure 6). As with the first and second steps, the center of the wafer (etch rate: 3592 Å/min) was etched faster than the left side (etch rate: 4233 Å/min), and the uniformity was relatively poor, at about 8.5%. However, since the third step over-etched a small number of targets, this level of uniformity was not considered problematic when sufficient over-etching was ensured.

### 3.5. Effect of Gas Chemistry: Selectivity and F/C Ratio

**Figure 7:**
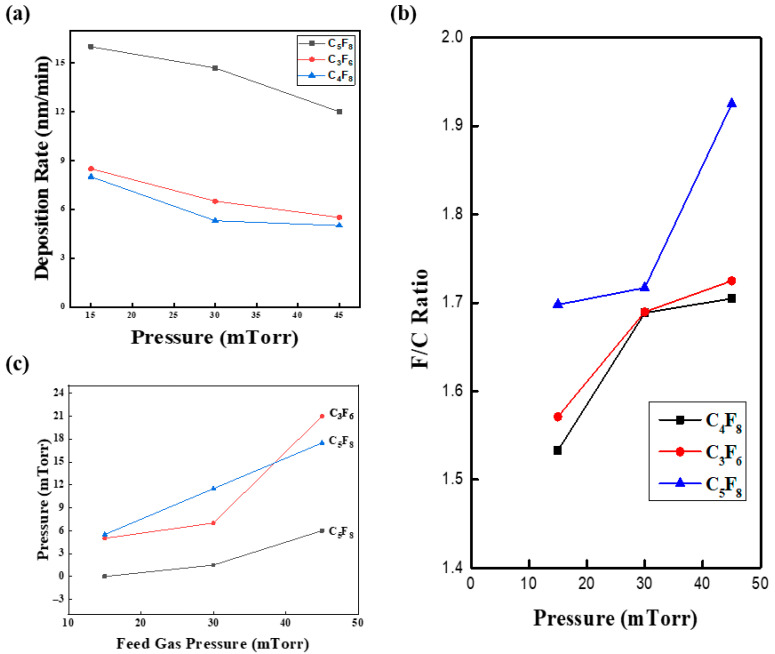
(**a**) Pressure dependence of fluorocarbon polymer deposition rate for each gas chemistry; (**b**) F/C ratio of the fluorocarbon polymer film; (**c**) partial-pressure variation of CF_4_ + C_2_F_6_ as a function of pressure.

The difference in selectivity between C_4_F_8_/O_2_/Ar and C_5_F_8_/O_2_/Ar arises from variations in the radical kinetics of polymer film deposition [23]. Generally, the etch characteristics of C_x_F_γ_ etchants are strongly influenced by the F/C ratio: higher F/C ratios yield lower selectivity and vice versa. However, the F/C ratio indicates only a general trend and does not signify that selectivity depends solely on etchant stoichiometry [24]. Interpretation should focus on radical kinetics after plasma ignition. Herein, we briefly review the plasma characteristics of the primary etchants (C_4_F_8_, C_5_F_8_, and C_3_F_6_) used in this study. Typically, the deposition rate of fluorocarbon polymers decreases as pressure increases, regardless of the etchant (Figure 7a). Moreover, the deposition rate of C_5_F_8_, which has a lower F/C ratio, is about twice that of other etchants.

However, an X-ray photoelectron spectroscopy (XPS) analysis of the deposited fluorocarbon polymer (Figure 7b) contradicts the stoichiometric interpretation. A simple evaluation based on the F/C ratio would suggest that the polymer formed from C_5_F_8_ plasma should have the highest carbon content; however, analysis shows that it actually possessed the highest fluorine content. This indicates that selectivity depends more on radical kinetics in the plasma than on stoichiometry. This conclusion is supported by an examination of the partial pressures of CF_4_ and C_2_F_6_ as a function of the feed gas pressure at a fixed RF power (Figure 7c). The partial pressures of CF_4_ and C_2_F_6_ increase with pressure, while the partial pressure rise for C_5_F_8_ is about half that of other etchants. Because CF_4_ and C_2_F_6_ are highly stable, their deposition reactions are limited, which results in lower selectivity compared with C_3_F_6_ or C_4_F_8_. Based on radical kinetics, fluorocarbon plasmas can be categorized into saturated fluorocarbons (e.g., C_4_F_8_) and unsaturated fluorocarbons (e.g., C_3_F_6_ and C_5_F_8_). Notably, the behavior of the parent gas and its plasma state should both be considered. Although C_3_F_6_ is an unsaturated fluorocarbon, its overall kinetics resemble those of C_4_F_8_. This can be explained by C_3_F_6_ transitioning from hexafluoropropene to hexafluorocyclopropane (c-C_3_F_6_) upon plasma ignition, exhibiting a behavior similar to C_4_F_8_ [25].

### 3.6. Difference in Polymer Deposition Characteristics Between C_4_F_8_ and C_5_F_8_ Gas Chemistry

**Figure 8:**
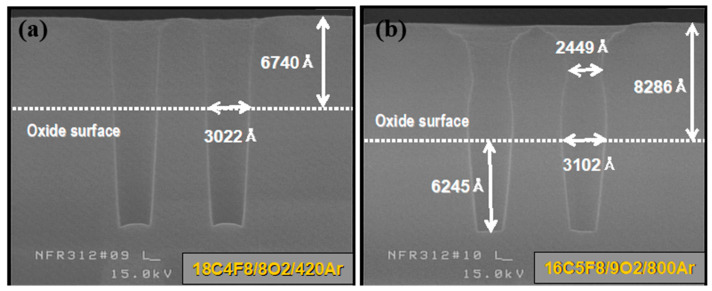
Results of analysis of PR loss and polymer deposition patterns according to etchant type. (**a**) 18 C_4_F_8_/8O_2_/420Ar and (**b**) 16 C_5_F_8_/9O_2_/800Ar.

Based on the details in the previous section, the deposition characteristics of fluorocarbon polymers with different etchants were evaluated. Figure 8 shows the PR loss after an M1CT etch using UV68,000 Å PR as a mask, comparing C_4_F_8_ and C_5_F_8_. With C_4_F_8_, the PR loss was about 1300 Å, while with C_5_F_8_, the PR thickness slightly increased relative to the initial coating. This indicates that with C_4_F_8_, stable molecules, such as CF_4_ or C_2_F_6_, are more actively generated in plasma than with C_5_F_8_; however, as these stable molecules have poor polymerization ability, their polymer redeposition rate is lower than the PR loss rate, which results in a net PR loss. This inference is supported by a comparison of the PR hole CD between the etchants. The oxide surface CD was similar (0.30–0.31 μm) in the first step, where CHF_3_/O_2_/Ar polymerization did not occur for either C_4_F_8_ or C_5_F_8_. However, after the second etch step, a bottleneck formed with C_5_F_8_, narrowing the CD to about 0.245 μm. Thus, with C_5_F_8_, polymer deposition increases as etching progresses, which indicates that polymer accumulates on the original PR.

### 3.7. Changes in Stopping Margin and CD Bias According to Over-Etch Target

**Figure 9:**
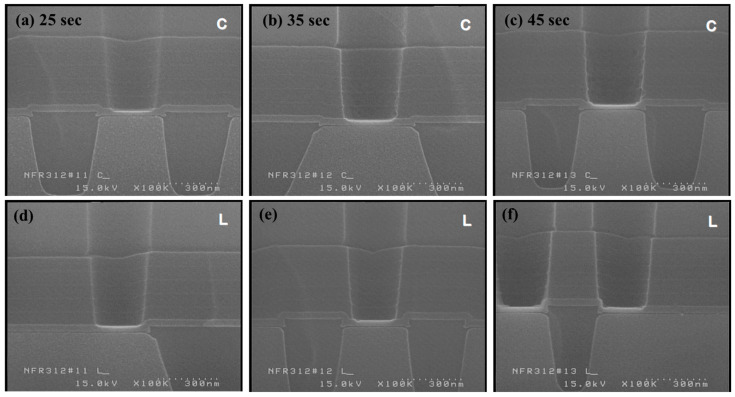
Over-etch target split evaluation for the third step. Even with increased over-etch time (25–45 s), BLC nitride loss remains nearly unchanged, demonstrating a stable stopping margin: (**a**) 25 s, center; (**b**) 35 s, center; (**c**) 45 s, center; (**d**) 25 s, left; (**e**) 45 s, left; (**f**) 25 s, left.

To more accurately evaluate the stopping margin under the 16 C_5_F_8_/9O_2_/800Ar condition, shown to be capable of stopping at 300 Å of BLC nitride in the third step, the margin was assessed using a target split (Figure 9). Rather than converting the exact etch target percentage into time, 10 s were incrementally added to the initial 25 s test condition. An SEM observation of the BLC nitride loss from the over-etch target splits showed no significant difference up to 45 s.

**Figure 10:**
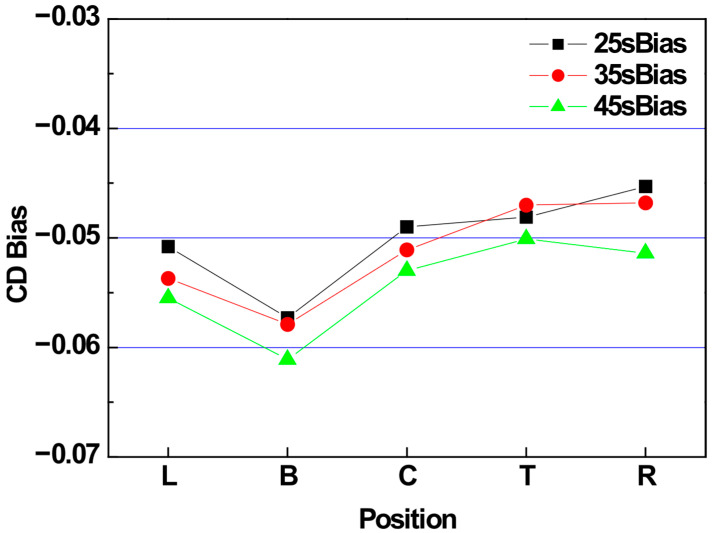
CD bias evaluation results based on the over-etch target split.

The CD bias trend across different over-etch targets was evaluated, as shown in Figure 10. Generally, as the over-etch target increases, the overall CD bias also increases. When polymer deposition on the sidewalls is minimal, an increased over-etch target typically leads to a reduction in CD bias. However, under conditions involving high oxide etch selectivity for nitride, active polymer deposition on the sidewalls produces a slope in the nitride film, thereby increasing the CD bias.

### 3.8. Process Margin According to Process Window Change

Since minor adjustments in the process window may not fully address established process conditions, the remnant nitride loss was evaluated after modifying the process window in the experiment. Due to the limited number of test wafers available, changes were restricted to the second-step etch target and the third-step process window. In the second step, an increase in the etch time (55 s → 65 s) reduced the stopping margin. Because small changes in gas flow rate can significantly affect etch stopping or selectivity, the gas ratio was adjusted in the third step to observe potential changes in the stopping margin or etch stop. Because test-wafer availability was limited, the experimental splits were designed using boundary-condition analysis rather than full matrix sampling. We focused on split conditions with a high likelihood of etch stopping (16 C_5_F_8_/9O_2_ → 17 C_5_F_8_/8O_2_) and a low stopping margin (16 C_5_F_8_/9O_2_ → 15 C_5_F_8_/10O_2_).

Among the three split conditions, this case—despite exhibiting the lowest selectivity and a higher second-step etch target—still demonstrated a favorable BLC nitride loss of less than 200 Å. The condition with the slightly reduced third-step selectivity and the condition with poor etch-stopping performance also demonstrated favorable process margins. In summary, increasing the second-step etch time (55 s → 60 s) and adjusting the third-step gas chemistry to a more favorable condition (16 C_5_F_8_/9O_2_ → 15 C_5_F_8_/10O_2_) for etch stopping were identified as optimal for the 3D integrated M1CT etch process margin.

**Figure 11:**
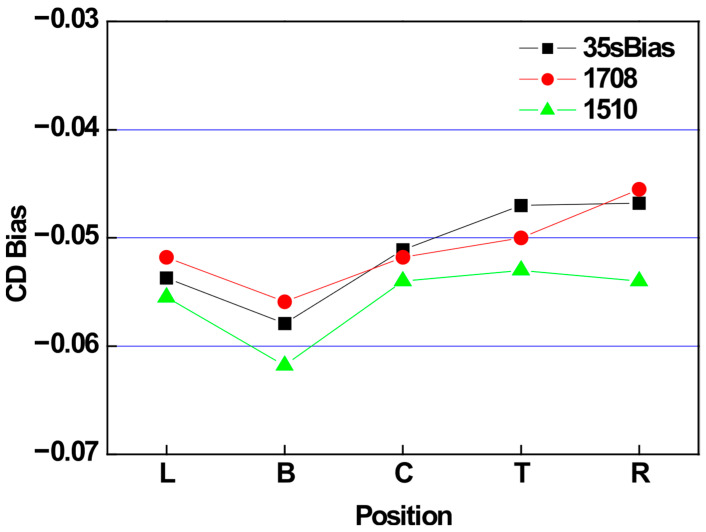
CD bias evaluation results according to process window changes.

The CD bias trend with respect to process window changes was evaluated, as shown in Figure 11. The 15 C_5_F_8_/10O_2_/800Ar condition with low selectivity showed a slight increase in CD bias, while the 17 C_5_F_8_/8O_2_/800Ar condition with high selectivity showed a decrease. This trend aligns with the previously observed CD bias changes associated with the over-etch target.

## 4. Conclusions

In this study, we investigated the metal-contact etching process tailored for 3D integration, focusing on the film-stack configuration and evaluating the resulting super-contact etching characteristics.

The total ILD thickness, excluding BLC nitride, was about 9400 Å, approximately 1000 Å higher than L180. An evaluation of the first-step etch showed a target thickness of about 2365 Å on the wafer’s left side, with a favorable bottom-rounded profile. In contrast, the center had a target thickness of about 2430 Å, with minimal micro-trench formation.

After the first-step etch to remove the liner TEOS and nitride, the stopping margins were evaluated under three conditions: (1) an L180 M1CT etch with a target thickness reduced by 22 s, (2) an M1CT baseline with ILD reduction, and (3) an increased C_5_F_8_/O_2_ ratio for improved selectivity and slightly reduced etch target thickness.

Under all three cases, the BLC nitride underwent punch-through, demonstrating that the conventional single-step etch scheme is insufficient for the thicker ONONO stack used in 3D integration.

To resolve this limitation, a three-step etch methodology was developed. By separating the main etch, profile-stabilization, and high-selectivity over-etch regions, the approach enabled improved selectivity control and stable nitride stopping behavior. The third-step over-etch maintained a robust stopping margin across various target splits and process-window variations while preserving acceptable CD bias—an advantage not achievable with conventional one-step etching.

Overall, the multi-step etch process demonstrates enhanced etch controllability, improved stopping-margin stability, and superior structural robustness, making it highly applicable to high-density DRAM, advanced logic devices, and other 3D-integrated architectures.

## Figures and Tables

**Table 1:** 3D integration M1CT ILD process flow.

	Process Flow
3D integration M1CT ILD	BLC nitride 300 Å → BPSG 3500 Å → TEOS 12,000 Å → 8000 Å after ILD CMP → Super-Via ILD & Si etch → Liner nitride 500 Å → Liner TEOS 1500 Å → Tungsten 1 μm deposition → Tungsten CMP → M1CT photo/etch

**Table 2:** 3D Int. M1CT ILD process flow. PE-nitride: plasma-enhanced nitride.

Recipe	Etch Rate (Å/min)		Etch Target			
	TEOS	PE-Nitride	TEOS 1400 Å	Nit 500 Å	Over-Etch	
17 mm/50/2200WSP/1400WBP/50CHF_3_/8.5O_2_/500Ar-23″	6240	7609	13.5 s	3.9 s	5.6 s	582 Å

**Table 3:** First-step etch target, over-etch percentage, and remnant ILD thickness monitoring.

Position	Total ILD Thickness	ILD2 Thickness	ILD2 Etch Target	Over-Etch (%)	Remnant ILD Thickness
Center	10,000 Å	1950 Å	2429 Å	24.5	7571 Å
Edge	9950 Å	1850 Å	2365 Å	27.8	7584 Å

**Table 4:** Optimized process conditions based on L180 M1CT etch for stopping-margin evaluation.

Step	Recipe	Remark
1	17 mm/50 mT/2200WSP/400WBP/50CHF_3_/8.5O_2_/500Ar/35T/10T–23″	Low selectivity
2	35 mm/30 mT/1900WSP/1600WBP/16C_5_F_8_/5CH_2_F_2_/21O_2_/800Ar/20T/10T–1′55″	Modified L180 M1CT etch condition
	35 mm/30 mT/1900WSP/1600WBP/16C_5_F_8_/5CH_2_F_2_/21O_2_/800Ar/20T/10T–1′30″	L180 B/L condition, etch target ↓
	35 mm/30 mT/1500WSP/1600WBP/16C_5_F_8_/19O_2_/800Ar/20T/10T–1′40″	Etch target ↓, selectivity ↑

**Table 5:** Second-step etch rate process conditions.

Step	Recipe
1	17 mm/50 mT/220WSP/1400WBP/50CHF_3_/8.5O_2_/500Ar/35T/10T-23″
2	20 mm/30 mT/2200WSP/1600WBP/18C_4_F_8_/15O_2_/420Ar/20T/10T-55″

**Table 6:** Third-step etch rate process conditions.

Step/Split Condition	Recipe
1/Baseline	17 mm/50 mT/50CHF_3_/8.5O_2_/500Ar/35T/10T–23″
2/Wafer 1	20 mm/30 mT/18C_4_F_8_/15O_2_/420Ar/20T/10T–55″
3/Wafer 1	20 mm/30 mT/18C_4_F_8_/8O_2_/420Ar/20T/10T–30″
2/Wafer 2	20 mm/30 mT/18C_4_F_8_/15O_2_/420Ar/20T/10T–55″
3/Wafer 3	35 mm/30 mT/16C_5_F_8_/9O_2_/800Ar/20T/10T–25″

## Data Availability

The original contributions presented in this study are included in the article. Further inquiries can be directed to the author.

## Abbreviations

The following abbreviations are used in this manuscript:

TEOS	Tetraethyl orthosilicate
CMOS	Complementary metal oxide semiconductor
CD	Critical dimension
SoC	System-on-chip
SiP	System-in-package
IC	Integrated circuit
M1	Metal 1
ILD	Interlayer dielectric
LBCTR	Left, bottom, center, top, and right
ONONO	Oxide–nitride–oxide–nitride–oxide
STI	Shallow trench isolation
M1CT	Metal-1 layer contact
L180	Logic baseline process integration
W	Tungsten
CMP	Chemical mechanical polishing
BPSG	Boro phospho silicate glass
BOE	Buffered oxide etch
SNC	Storage node contact
BLC	Bit-line contact
DRAM	Dynamic random access memory
FICD	Final inspection critical dimension
PR	Photo resist
SEM	Scanning electron microscopy
PE-TEOS	Plasma-enhanced tetraethyl orthosilicate
MFC	Mass flow controller
XPS	X-ray photoelectron spectroscopy
